# PTZ Camera-Based Displacement Sensor System with Perspective Distortion Correction Unit for Early Detection of Building Destruction

**DOI:** 10.3390/s17030430

**Published:** 2017-02-23

**Authors:** Yoosoo Jeong, Daejin Park, Kil Houm Park

**Affiliations:** School of Electronics Engineering, Kyungpook National University, Daegu 41566, Korea; ysjung@ee.knu.ac.kr

**Keywords:** structural health monitoring, displacement measurement, non-contact measurement, vision-based system, PTZ camera, perspective transformation

## Abstract

This paper presents a pan-tilt-zoom (PTZ) camera-based displacement measurement system, specially based on the perspective distortion correction technique for the early detection of building destruction. The proposed PTZ-based vision system rotates the camera to monitor the specific targets from various distances and controls the zoom level of the lens for a constant field of view (FOV). The proposed approach adopts perspective distortion correction to expand the measurable range in monitoring the displacement of the target structure. The implemented system successfully obtains the displacement information in structures, which is not easily accessible on the remote site. We manually measured the displacement acquired from markers which is attached on a sample of structures covering a wide geographic region. Our approach using a PTZ-based camera reduces the perspective distortion, so that the improved system could overcome limitations of previous works related to displacement measurement. Evaluation results show that a PTZ-based displacement sensor system with the proposed distortion correction unit is possibly a cost effective and easy-to-install solution for commercialization.

## 1. Introduction and Related Works

Displacement measurement is an important technique required in diverse industries, such as construction, machinery, and robotics. In particular, displacement measurement of critical structural positions in large structures is a vital component of structural health monitoring (SHM). Extensive research has been conducted in the field of displacement measurement, including how well structures are designed to effectively respond to external disturbances such as earthquakes and heavy winds, as well as the level of structural risk that could lead to a building collapsing [1].

Existing contact-type sensors designed to measure displacement have limitations. For example, displacement measurement of large structures, such as a bridge, is difficult to measure due to installation challenges at critical structural positions [2], limitations in the measurement of maximum displacement [3], and limitations in establishing stationary reference points near the structure [4]. In addition to the technical aspects, existing displacement measurement systems also suffer from high costs and a short life-cycle, which limits the cost effectiveness of such systems.

Emerging research is focused on displacement measurement technologies that rely on cameras instead of contact-type displacement sensors to measure structural displacement. This is accomplished by using vision-based measurement technologies that recognize and measure the displacement of markers attached at critical structural positions.

An effective SHM system includes displacement measurements at both low and high frequencies. At extremely low frequencies, displacement measurement focuses on the excess lag and the load of a structure over time, while at high frequencies, displacement measurement is focused on structural stress caused by external, environmental disturbances, such as earthquakes or heavy winds. While SHM is concerned with displacement at either frequency, monitoring at low frequencies should take priority, as the critical points of a structure can move slowly over time and monitoring at an extremely low frequency can provide early warning symptoms of building collapse, hence preventing such a collapse from occurring. In this respect, a previous approach measuring displacement at an extremely low frequency through a vision-based displacement measurement system was introduced, so that the risk of a bridge collapsing could be detected in advance [5].

The accuracy of vision-based displacement measurement technology is the most critical aspect for commercialization, which is at the core of recent displacement measurement research [6] by statistically considering uncertainty due to various effects, including camera calibration. An advanced template matching was applied to increase the accuracy of vision-based displacement measurement, which unlike conventional template matching, determined the detection of objects with sub-pixel accuracy [7]. Sladek showed that the accuracy of displacement measurement can be improved by correcting for the camera lens distortion and scale distortion [8]. Meanwhile, a previous study [9] showed that the accuracy of displacement measurement could be increased by using the random sample consensus (RANSAC) algorithm to accurately extrapolate two orthogonal lines drawn on the marker.

Moreover, Fukuda developed a stand-alone vision-based displacement measurement system, based on a connected laptop and camcorder, which allowed displacement measurement to be achieved through a wireless Internet connection and further reinforced the cost and installation advantages of vision-based displacement measurement systems [10].

Outside of these studies, Park [1] and Lee [11] successfully applied a vision-based displacement measurement system to high-rise buildings [1], while Kim developed a video-based algorithm to measure the tension of hanger cables connecting a bridge to the pier [2].

The latest advances of vision-based sensors and their applications are introduced. A cost effective vision-based structural health monitoring approach has been presented [12], in which the displacement is accurately measured by considering the tilt between the target and marker. The cable tension measurement based on a vision-based sensor was successfully introduced [13].

Despite these advantages, conventional studies on vision-based displacement systems have relied on images generated from a fixed viewing angle camera, which necessitated a trade-off between the field of view (FOV) and spatial resolution. In other words, any effort to increase the FOV by raising the number of markers resulted in a decrease in spatial resolution. Similarly, any effort to increase the measurement accuracy by increasing the spatial resolution resulted in a lower number of markers and decreased FOV. Hence, conventional studies on displacement measurement relied on 1–2 markers per camera to measure displacement in large structures or focused on smaller structures for experiments.

The existing methods have limitations in terms of measurable range because they are based on the fixed camera with the narrow and constant FOV. This paper proposes a vision-based displacement measurement system based on a pan-tilt-zoom (PTZ) camera, which can extend the measurable range on monitoring displacement.

To perform an early estimation of collapse risk for a building structure, we assume that displacement happens at very low frequency and the positioned markers slightly move along the plane on which markers are attached. Markers’ displacement will be monitored sequentially using visual information described on markers.

The algorithm underlying the PTZ displacement measurement system can be explained as follows. First, template matching is applied to find the region of interest (ROI) of the marker.

Although we adopt the RANSAC ellipse fitting-based model with coordinates of marker contours in the ROI, the center point of the marker is detected in sub-pixel accuracy. As it relates to the accuracy of displacement measurement, the center point is mapped to the perspective distortion correction plane which we call the standard plane.

This paper is organized as follows: Section 2 describes a displacement measurement system based on a PTZ camera and explains the algorithm underpinning our proposed system; Section 3 describes the experimental results on diverse structures; Section 4 concludes the paper.

## 2. Proposed Architecture

**Figure 1:**
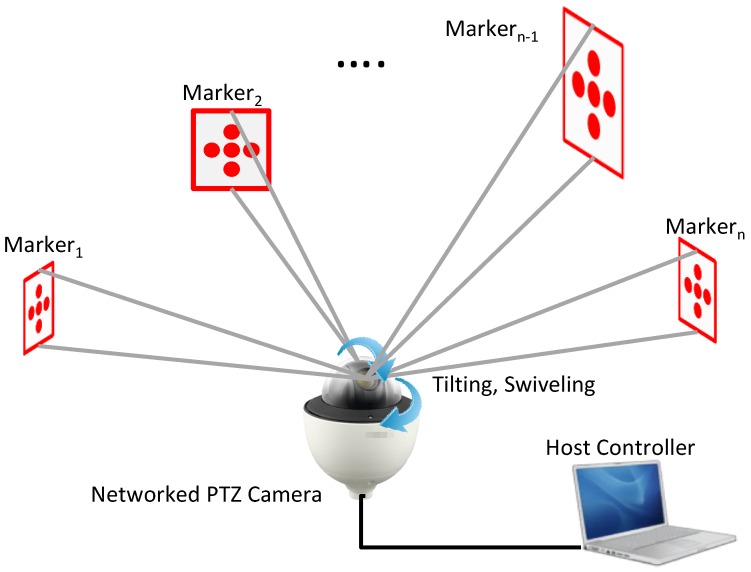
Conceptualization of a PTZ camera-based displacement measurement system.

Figure 1 shows the overall architecture of a PTZ camera-based displacement measurement system (PTZ system). As shown, a PTZ system is comprised of an internet protocol (IP) camera, various markers, and a computer to process the video images. To improve the installation process, we suggest using an IP camera with PTZ functionality, which allows the video from the camera to be fed wirelessly to a PC. Once the video is received, displacement can be measured by applying an algorithm to process the video images.

**Figure 2:**
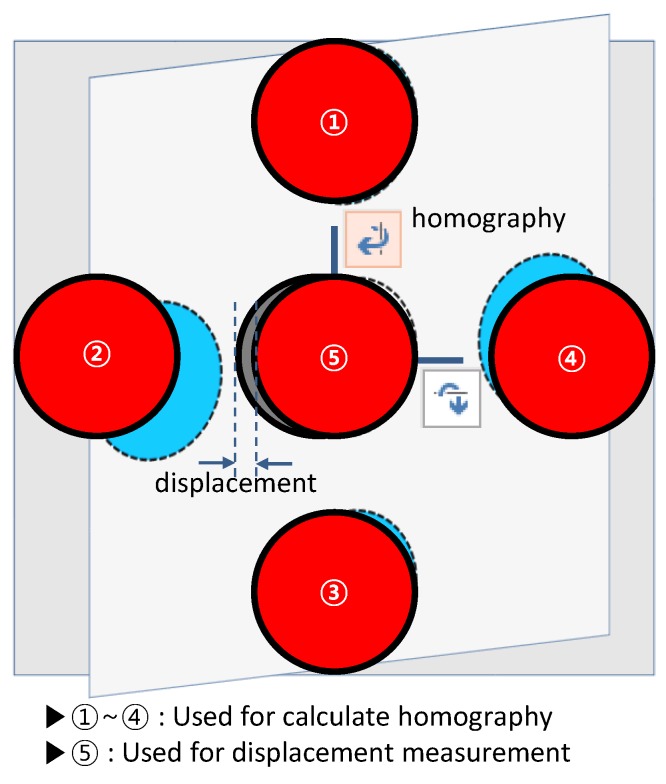
A marker for a PTZ camera-based displacement measurement system.

As shown, each marker consists of five red circles based on information acquired from the camera related to the attached marker plane. As represented in Figure 2, the center points of the four outer circles ①–④, which are considered as feature points, are used to calculate a homography center point (see the fourth step in Section 2.2 for a detailed description of this process); the center point of ⑤ is used for measuring displacement.

This paper proposes an algorithm for a PTZ system that can largely be divided into two key processes. The first process involves constructing preset data based on diverse information collected in advance on marker images and PTZ marker coordinates needed to measure displacement. The second process then utilizes the preset data to film the markers and measure the displacement. Section 2.1 describes the process of constructing preset data while Section 2.2 describes the process of sequentially measuring the displacement of markers.

### 2.1. Constructing Preset Data

**Figure 3:**
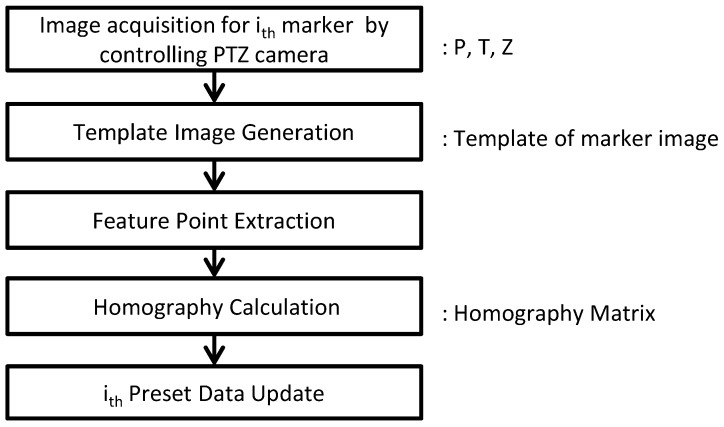
Flow chart depicting the construction of preset data.

Constructing preset data is achieved through the following steps. First, the measurable plane for a marker is located by manually using the pan-tilt-zoom functions of the PTZ camera, with the relevant PTZ coordinates then entered into the preset data. Second, a specific minimum bounding rectangle (MBR) based on the marker, has to be manually registered by the administrator monitoring the target building. Third, feature points are extracted from the template image with the central point of the four most outer points, which are used to eliminate perspective distortion. Finally, from this, a homography is calculated, for which the details are explained in Section 2.1.4. Figure 3 depicts the flow of steps used in constructing preset data.

**Figure 4:**
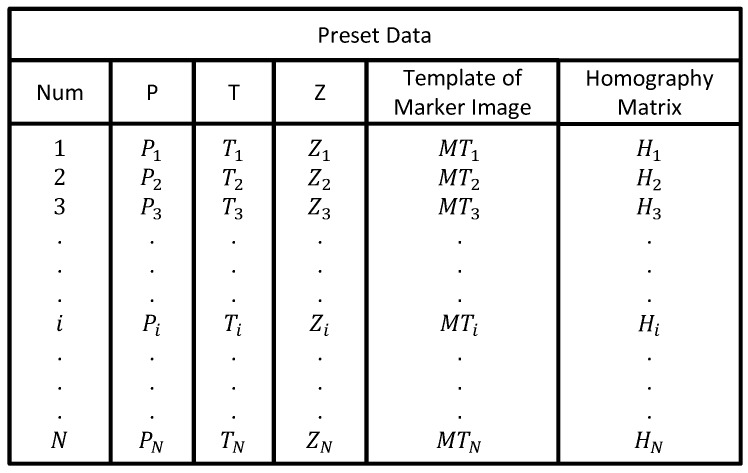
Example of representing preset data.

The result of applying the process shown in Figure 3 is a set of preset data to the *i*-th marker. Figure 4 shows the organization of preset data to the *i*-th marker with $P_{i}$, $T_{i}$, $Z_{i}$ representing the PTZ coordinates, $MT_{i}$ representing the template image, and $H_{i}$ representing the homography matrix.

#### 2.1.1. Step 1. Obtaining P,T,Z Coordinates of the *i*-th Marker

**Figure 5:**
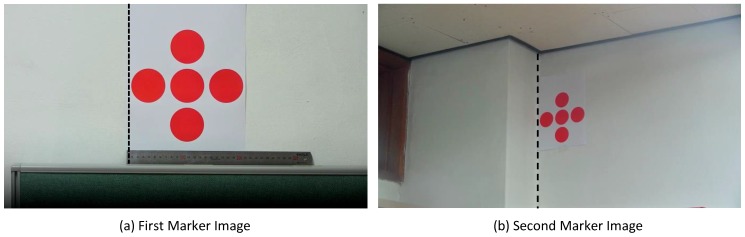
Example of acquiring a marker image to set PTZ coordinates.

The first step in constructing the preset data is entering the PTZ coordinates of the *i*-th marker from relevant marker images. This is achieved by manually controlling the PTZ camera to locate a measurable marker plane, and then entering the PTZ coordinates of the location in the preset data as $P_{i}$, $T_{i}$, $Z_{i}$, respectively, as depicted in Figure 5.

#### 2.1.2. Step 2. Generating the Template Image

**Figure 6:**
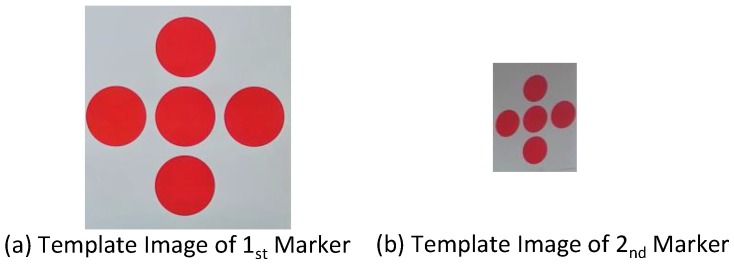
Template images.

Step two in the process involves taking the marker images obtained in the first step and manually creating MBR based on the background and domain of the markers, which are then entered into the preset data as template images for the *i*-th marker ($MT_{i}$). As explained in Section 2.2, the template images will be used for template matching to find the ROI of the markers. Figure 6 depicts template images acquired by manually setting the MBR for images shown in Figure 5.

#### 2.1.3. Step 3. Extraction of Feature Points

Based on template images created in step two, an algorithm to extract the central point of the four most outer points is applied for step three. This technique is explained in the following two methods: through the Otsu threshold method and RANSAC ellipse fitting.

A Otsu threshold method

The first step in detecting the central point of the marker begins with extracting five circles in the MBR and converting the MBR to gray-scale. The next step is executing the Otsu algorithm to determine the threshold value [14].

**Figure 7:**
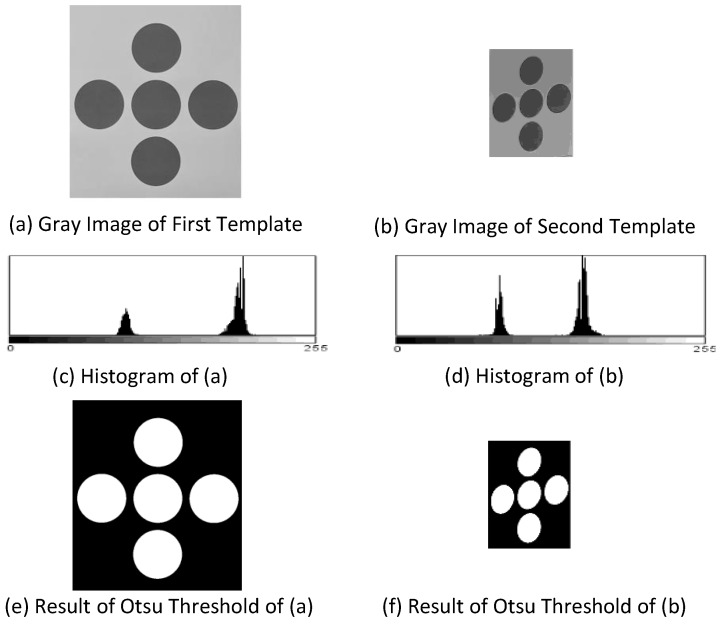
Process of the Otsu threshold method.

This process is depicted in Figure 7. Firstly, Figure 7a,b show the gray-scale conversions of Figure 6. Next, as shown in the histograms represented in Figure 7c,d, the gray-scale images have two main distributions. The performance effectiveness of the segmentation is directly related to how the threshold value is determined considering these two main distributions, which we suggest can best be determined by applying the Otsu threshold method.

For the case of the initial threshold value ‘*T*’, the measured pixels are categorized by the following: *α* is the ratio of darker pixels rather than *T*, $\mu_{1}$ is the average brightness for their pixels, $\sigma_{1}^{2}$ is its variance. *β* is the ratio of brighter pixels rather than *T*, $\mu_{2}$ is the average brightness for their pixels, $\sigma_{2}^{2}$ is its variance. The Otsu method selects the threshold value ‘*T*’ to maximize inter-class variance $\sigma_{T}$, which is described in the following equation:

(1) $$\sigma_{T}=\alpha \beta {\left(\mu_{1}-\mu_{2}\right)}^{2}$$

B RANSAC ellipse fitting

The next step in detecting the feature points in preset data is modeling for ellipse, which involves finding the center point for each of the five circles in the MBR. The MBR for the marker is detected by the result of the template matching algorithm in Section 2.2. Notably, the center point of the central circle is used for detecting displacement, while the center points of the remaining circles are used to calculate the homography matrix. The ellipse equation and center point ($c_{x},c_{y}$) are generally described in Equations (2) and (3). The coefficients of ellipse Equation (2) represent ellipse parameters, which are calculated by using the least square solution technique. In this paper, we could determine the optimal value of the ellipse parameters by using the RANSAC ellipse fitting algorithm [15].

(2) $$ax^{2}+bxy+cy^{2}+dx+ey+f=0$$

(3) $$c_{x}=\frac{2cd-be}{b_{2}-4ac},c_{y}=\frac{2ae-bd}{b_{2}-4ac}$$

**Figure 8:**
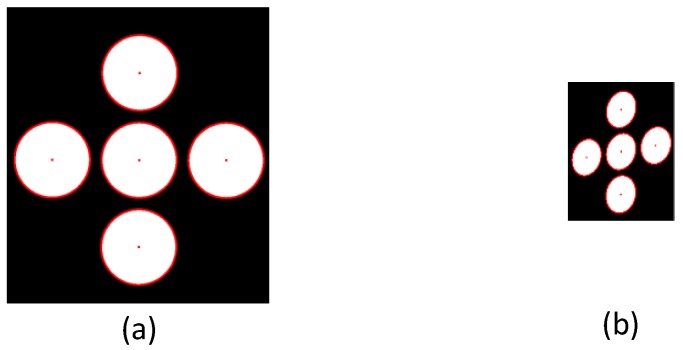
Results of optimal ellipse fitting.

Figure 8 depicts an optimal ellipse model derived by applying the RANSAC ellipse fitting algorithm to the contour points of the circles in Figure 7e,f. We first obtained a binary image by the Otsu method, then we selected a contour in which a pixel value is 255 and which has a minimum of two pixels with the value of zero in eight neighborhoods. The centers of the ellipse model and outside marker coordinates are highlighted in red. Hence, an optimal ellipse model can be determined by applying the RANSAC algorithm of ellipse fitting. This optimizes the model by excluding invalid data points, an important advantage in situations where a portion of the data may be unreliable due to some area of the circle either closing or showing an unclear edge.

We first construct an ellipse model by using all extracted contour points, and determine the optimal coefficients through the RANSAC Ellipse Fitting algorithm. By applying the RANSAC method, outliers become contour coordinates of the other four circles, therefore we could construct an ellipse model for a specific circle in the target marker. These procedures are iteratively applied for five circles to construct the ellipse models. Finally, we use the center points, which are extracted from these five elliptic models, as feature points.

The five extracted feature points need to be labeled to determine which points correspond with the expected references for homography calculation and displacement measurement later. We labeled four points with smallest *y*, smallest *x*, largest *y*, largest *x*, and the last remaining point as 1st, 2nd, 3rd, 4th, and the 5th featured point, respectively. The 1st~4th points are used as a reference to calculate homography, and the 5th point is mapped into the standard plane to perform displacement measurement.

The circles on the target wall are projected as ellipse on the camera-captured plane. This is why we extract the feature point by modeling the circle of the marker as ellipse. This approach results in a more reliable characterization of the tilt relationship between the marker plane and the projected plane. In addition, this approach effectively selects data by eliminating the outlier, therefore the center position can be reliably determined under the situation in which the edge is unclear or partially covered by other obstacles. We could measure the center position at sub-pixel resolution by using only the inlier in this paper.

#### 2.1.4. Step 4. Determining Homography

The target plane, on which the marker is attached, is not parallel with that on the camera sensor. That is why the perspective distortion in the captured image is inevitable. The displacement of the marker on the target plane has to be measured after the perspective distortion effect is compensated by considering the locational relationship between two planes.

**Figure 9:**
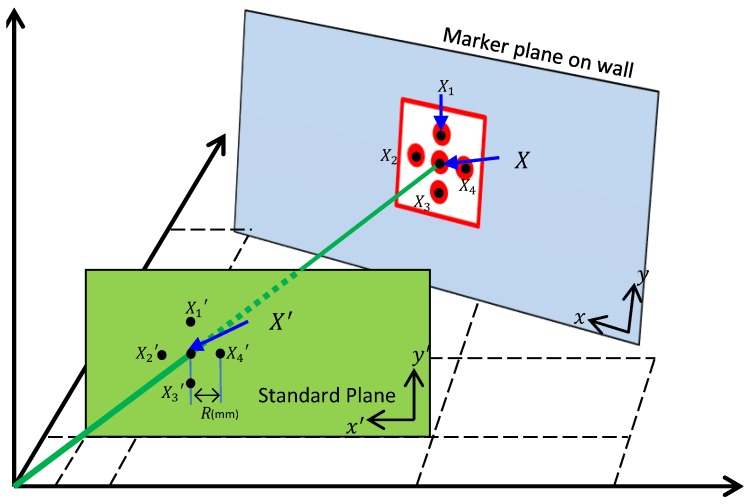
Concept of homography.

In this paper, for accurate measurement of the marker displacement, we adopted homography to eliminate the perspective distortion. As shown in Figure 9, a homography is the result of mapping the two planes by converting the *X* point on the *x*-*y* plane into a newly-projected point $X^{\prime }$ on the $x^{\prime }$–$y^{\prime }$ plane along a straight line starting from the origin.

The points $X_{1}\left(x_{1},y_{1}\right),X_{2}\left(x_{2},y_{2}\right),X_{3}\left(x_{3},y_{3}\right)$, and $X_{4}\left(x_{4},y_{4}\right)$ are located on the *x*–*y* plane, and these correspond to the points $X_{1}^{\prime }\left(x_{1}^{\prime },y_{1}^{\prime }\right),X_{2}^{\prime }\left(x_{2}^{\prime },y_{2}^{\prime }\right),X_{3}^{\prime }\left(x_{3}^{\prime },y_{3}^{\prime }\right)$, and $X_{4}^{\prime }\left(x_{4}^{\prime },y_{4}^{\prime }\right)$ on the $x^{\prime }$–$y^{\prime }$ plane, respectively. Specially, the points $X_{1}^{\prime },X_{2}^{\prime },X_{3}^{\prime }$, and $X_{4}^{\prime }$ are located at the *R* (millimeter scale) distance away from origin $X^{\prime }$; the distance *R* from these four points is the same, and they intersect at 90${}^{\circ }$, still following the property of the orthogonal coordinates system. Therefore, the standard plane can be defined as the perspective distortion-corrected plane in millimeter unit through homography.

Homography is a linear transformation which can be expressed through the following equation:

(4) $$\left[\begin{array}{c} x_{i}^{{}^{\prime }}\\ y_{i}^{{}^{\prime }}\\ 1^{{}^{\prime }} \end{array}\right]=H\left[\begin{array}{c} x_{i}\\ y_{i}\\ 1 \end{array}\right]$$

wherein *H* is a 3×3 matrix, then matrix *H* can be calculated through the direct linear transformation (DLT) algorithm [16].

In this paper, a minimum of four points is needed for the calculation of the homography matrix to map the marker plane to the standard plane by applying the direct linear transformation (DLT) algorithm [17]. *R* in Figure 9 is actual distance in millimeters (mm) between the center points in the target markers. The manner of millimeter-scale measurement requires statistical consideration for uncertainty due to various effects [6].

The homography is determined through a mapping process using the DLT algorithm, based on *x*–*y* coordinates of the pre-converted image and $x^{\prime }$–$y^{\prime }$ coordinates of the converted image. This causes the actual distance in millimeters (mm) between the four outer angles of the marker at the four vertex points for each side of the MBR. Thus, it becomes the distance unit in millimeters of the plane of the standard plane. The calculated homography matrix *H* is entered into the preset data as $H_{i}$.

### 2.2. Description of Processing Algorithm

**Figure 10:**
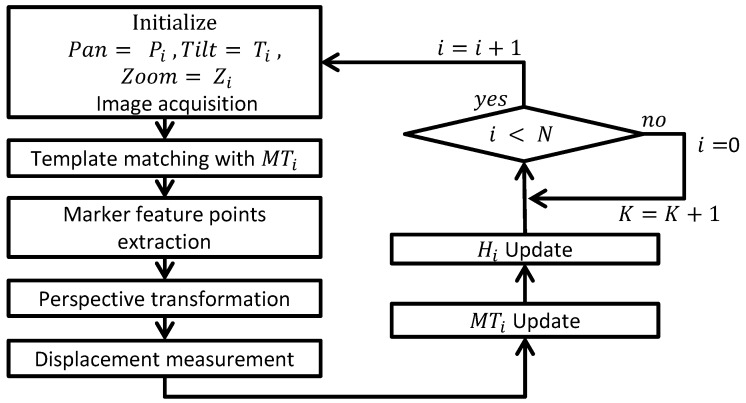
Flow chart of the displacement processing algorithm.

This paper proposes that displacement can be measured by applying an algorithm to PTZ camera images—a six-step sequential process to measure *N* displacement. This process is depicted in Figure 10.

#### 2.2.1. Step 1. Image Acquisition $MT_{i}$

**Figure 11:**
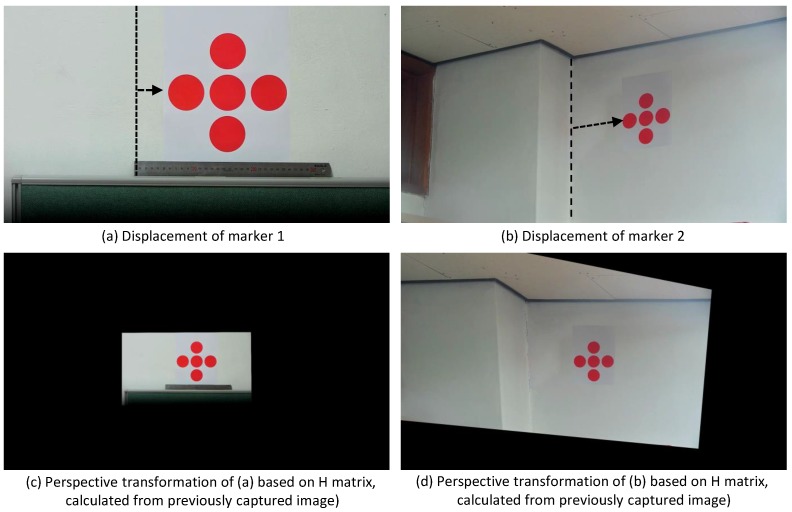
Image results from the perspective transformation process.

As explained earlier in Section 2.1, related to constructing preset data, image acquisition and template matching begins by automatically setting the PTZ coordinates of the *i*-th marker from relevant marker images. In Figure 11a,b both images are acquired in processing parts. This is achieved by automatically setting the PTZ coordinates of the location in the preset data as $P_{i}$, $T_{i}$, $Z_{i}$, respectively. The displacement on the marker is represented by an arrow in Figure 11a,b. Figure 11c,d are the result of perspective transformation, which are done by the calculation of the H matrix from the images captured in the previous level for Figure 11a,b respectively.

#### 2.2.2. Step 2. Template Matching with $MT_{i}$

Based on the marker images, the ROIs are found by template matching with a template image, which is stored in preset data $MT_{i}$ [18]. The template-based matching we used is basically performed by the coefficient-map calculation between the template image and the target image, which is captured through the sliding window. The coefficient map (CM) calculation method we adopted is described with the following equation for the squared difference:

(5) $$CM\left(x,y\right)=\sum_{x^{\prime },y^{\prime }}^{}{\left(T\left(m,n\right)-I_{i}\left(x+m,y+n\right)\right)}^{2}$$

$I_{i}$ is an $i_{th}$ acquired marker image and (x,y) is a coordinate in the template image. *T* is the template image in which the position (m,n) is located. The smaller CM value means that two images in the current-sliding window are similar, so that the pixel with the smallest CM value is selected as a top left point, and its rectangular, which is the same size as the template image, is used as the MBR of the marker.

#### 2.2.3. Step 3. Feature Points Extraction for Marker

See the Section 2.1.

#### 2.2.4. Step 4. Perspective Transformation

The displacement of the marker on the target wall must be calculated by projecting the marker plane into the perspective distortion-corrected plane as a standard plane. The first step in calculating the displacement involves finding the center point $\left(x^{\prime },y^{\prime }\right)$ of the marker on the standard plane, which is achieved by applying perspective transformation with an element of H denoted as $h_{11}$, $h_{12}$, .. $h_{33}$ respectively; and the center point of the marker on the *x*–*y* plane is represented as the ellipse center point (x,y) in the following equation:

(6) $$x^{{}^{\prime }}=\frac{h_{11}x+h_{12}y+h_{13}}{h_{31}x+h_{32}y+h_{33}},y^{{}^{\prime }}=\frac{h_{21}x+h_{22}y+h_{23}}{h_{31}x+h_{32}y+h_{33}}$$

#### 2.2.5. Step 5. Displacement Measurement

The displacement measurement in the current level is based on the *H*-matrix as a statically determined constant table, which is calculated in the previously captured stage. Starting from the center point $X^{\prime }$, which is a center of the marker in the previous stage, the vector to the new center point $\left(x^{\prime },y^{\prime }\right)$ in the current level is represented by the displacement. The total displacement measurement can be done by accumulating offsets from previously measured *x*, *y* coordinates to the *x*, *y* coordinates of the marker. The magnitude of the determined displacement ($MD_{x}$) in the *x*-direction and ($MD_{y}$) in the *y*-direction can be represented by the following two equations:

(7) $$MD_{x}=\sqrt{{\left(\sum_{t=1}^{K}\left(x^{\prime }\left(t\right)-X_{x}^{\prime }\left(t\right)\right)\right)}^{2}}$$

(8) $$MD_{y}=\sqrt{{\left(\sum_{t=1}^{K}\left(y^{\prime }\left(t\right)-X_{y}^{\prime }\left(t\right)\right)\right)}^{2}}$$

The magnitude of the total displacement (*MD*) can be represented by the following equation:

(9) $$MD=\sqrt{{\left(\sum_{t=1}^{K}\left(x^{\prime }\left(t\right)-X_{x}^{\prime }\left(t\right)\right)\right)}^{2}+{\left(\sum_{t=1}^{K}\left(y^{\prime }\left(t\right)-X_{y}^{\prime }\left(t\right)\right)\right)}^{2}}$$

wherein *K* means the number of rounds in capturing the image; $x^{\prime }\left(t\right),y^{\prime }\left(t\right),X_{x}^{\prime }\left(t\right),X_{y}^{\prime }\left(t\right)$ correspond to $x^{\prime },y^{\prime },X_{x}^{\prime },X_{y}^{\prime }$ in the $t_{th}$ captured image.

In addition, the following equation is an example of the *H* matrix that we used for the first marker (marker 1).

(10) $$H_{1}=\left[\begin{array}{ccc} 0.32991 & -0.00316183 & 636.29\\ -0.00326231 & 0.330228 & 397.409\\ -0.0000037836 & -0.00000521451 & 1 \end{array}\right]$$

#### 2.2.6. Step 6. Updating the $MT_{i}$

We register the extracted ROI image as a template image $MT_{i}$, which is the result of the template-matching step for the currently-monitored marker. This process is necessary to prevent any failure to detect the marker, given that the camera’s projected shape of the marker could change from the marker shifting, or the template-matching rate could fall due to illumination changes.

#### 2.2.7. Step 7. Updating the $H_{i}$

The homograph matrix, which is used to transform the target plane of a marker into the standard plane, has to be recalculated. The recalculated homography $H_{i}$ is updated to the *i*-th marker and the displacement algorithm process finishes.

The feature point extraction based on the Otsu threshold method is less sensitive according to consistent illumination fluctuation over all the circles of the marker surface. Local variation of light level on markers affects the threshold level change on a specific segmented region. Perspective distortion of the marker always results in an ellipse circle regardless of the direction from which the camera captures image, thus this assumption helps efficient feature extraction from the distortion information.

## 3. Experimental Results

The purpose of this paper was to verify the performance effectiveness of a displacement measurement system based on images generated by a PTZ camera. We evaluate the proposed system on markers moving at a low frequency to observe the accuracy of displacement measurements. Markers used in the tests are shown in Table 1.

**Figure 12:**
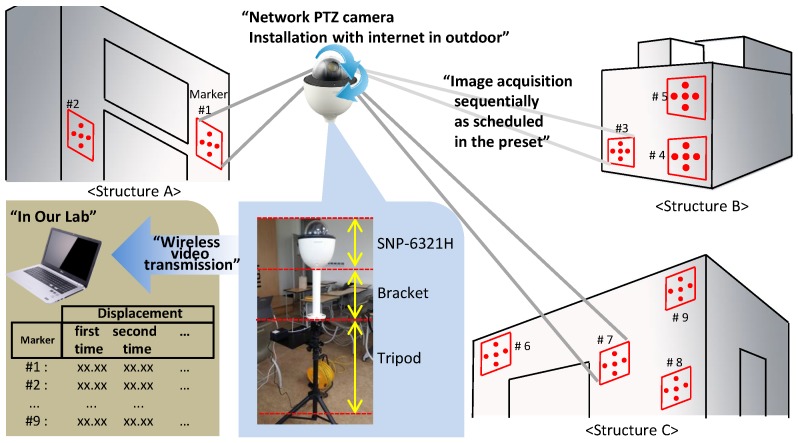
Experimental setup.

The test environment setup is described in Figure 12. The nine markers are distributed on the several structures, which are at various distance. The PTZ-camera rotates its angle, sequentially captures the markers and transmits the projected image via a wireless channel. A Hanwha Techwin PTZ camera (model SNP-6321H) was used to capture marker images, for which our proposed software performs the image processing algorithm in the host side, which is implemented using C/C++ programming language. The SNP-6321H model has a CMOS-type sensor, with a maximum resolution of 1920 × 1080 pixels and a video transmission rate of 30 frames per second (FPS), and the focal length of the optical lens varies in the range of 4.44~142.6 mm with 32× optical zoom and automatic focus. The angle of view (AOV) is 62.8${}^{\circ }$ (Wide)~2.23${}^{\circ }$ (Tele) in the horizontal direction and 36.8${}^{\circ }$ (Wide)~1.26${}^{\circ }$ (Tele) in the vertical direction. The camera SNP-6321H is installed on a tripod to monitor three buildings with nine markers. The PTZ camera sequentially obtains the images by rotating its angle and zooming, with the PTZ coordinates in preset data, to monitor the other markers; it then wirelessly transfers the data to the host computer.

The camera can acquire an image at 30 frames per second for each marker, but in this experiment, the camera captures 10 images and transfers them to the host computer in one second by considering the bandwidth of wireless communication. This means that the allowed time to perform the analysis for one image is 100 ms. Our implemented system could perform the entire algorithm for displacement measurement at 73 ms processing time. We performed the analysis for 10 captured images for each marker per second, and the rotation of the camera angle takes one second, therefore the processing time for each marker is 2 s. The sequential analysis for the other markers is iteratively performed by rotating the camera. Therefore, one round of analysis for all nine markers, which is described in Table 1, takes a total of 18 s. We perform a total of 10 rounds of analysis for the experiment in Table 2.

**Figure 13:**
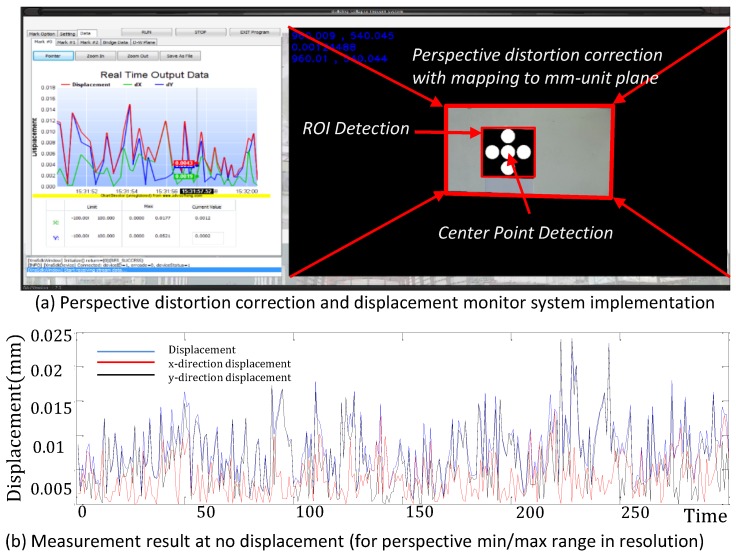
Host interface to monitor displacement in real time.

The tests were conducted on nine markers with distances from the camera between 5 m and 72 m, by capturing the image using H.264 codec at 1920 × 1080 resolution, as shown in Table 1 and Table 2. Each marker was printed on 210 mm × 297 mm (A4-size) paper, which consisted of five red circles, spaced 69 mm from the center. The results on markers 1–9 are shown below. We measured the trajectory of displacement during a specific amount of time. Figure 13a shows the implemented host interface to monitor the displacement of a target marker in real time including measurement result in case of no target marker movement, as shown in Figure 13b. After the user tries to select the target marker and manually focus on the monitored region, the internal detection algorithm performs the detection of the center position, feature extraction, and perspective distortion correction. As shown in the left of Figure 13a, we first check the amount of error in the case of no displacement to verify the experiment environment, including stable camera installation. Figure 13b shows a result of long-term displacement measurement.

As shown in Figure 13b, the error in the *y*-direction is more severe than that in the *x*-direction. The marker image, which is installed on the target wall, is projected into the another plane of the camera. This causes spatial distortion in terms of the position of the projected circles because the image sensor in the camera spatially captures the circle image at the different resolution relatively. In this case, in terms of the distorted angle, the error of displacement, which is measured in the *y*-direction, is slightly larger than a result in the *x*-direction.

**Figure 14:**
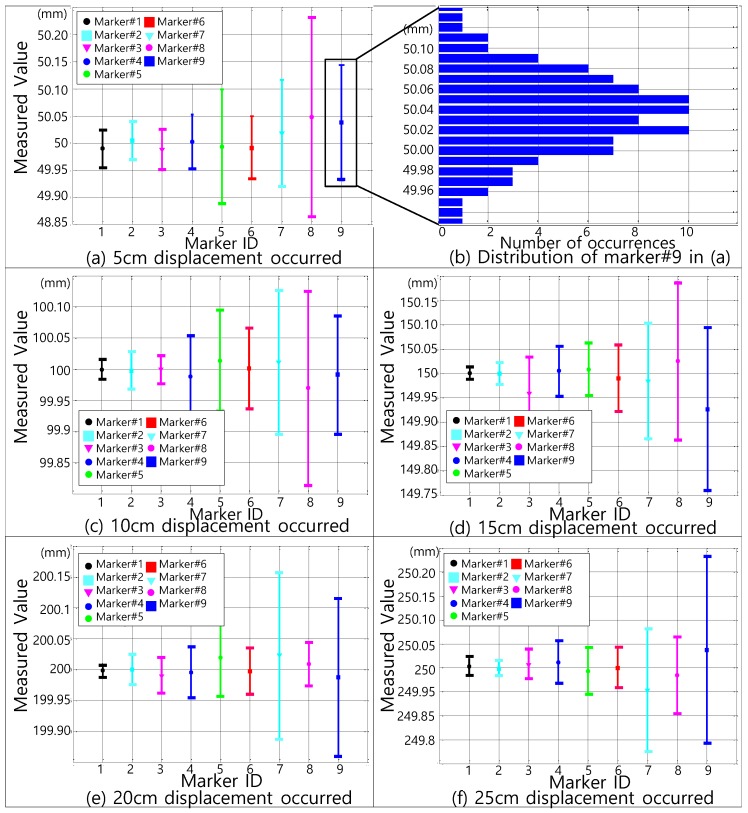
Displacement measurement results according to the markers.

Figure 14 shows the measurement results of the displacement for each marker at different distances and angles. The evaluation results show that our approach shows small variation with respect to the distance between the camera and markers.

The evaluation results are summarized as follows:

A Though the distance between the camera and target marker increases, spatial resolution variation, as a result of displacement measurement, is relatively small

The existing vision-based displacement measurement approaches using a lens with fixed magnification result in large degradation of spatial resolution according to the increase of distance between the camera and marker. Consequentially, they require more accurate lens specification to cover maximum distance, or to extend the measurable range. Because our approach could utilize a specific zoom level and lens angle based on the PTZ camera considering the marker location for multiple markers, our implemented system results in stable spatial resolution although the distance is largely increased, as shown in Table 2.

The measured displacement, which is multiple captured, is statically represented in terms of *RMS* with the following equation:

(11) $$RMS=\sqrt{\frac{\sum_{i=1}^{n}{\left(MD_{i}-GT\right)}^{2}}{n}}$$

$MD_{i}$ is $i_{th}$ measured displacement, the ground truth (*GT*) means the actual displacement value that we manually moved as a reference, for which 5 cm, 10 cm, 15 cm, 20 cm, and 25 cm are used for our experiment in Figure 14. The number of measurements for each *GT* is 100.

B Extend the measurable range by monitoring the target at a slightly slower sample rate

As explained, we adopted a PTZ-camera-based vision sensing approach to monitor markers, which are installed in various locations. Our approach enables to extend the measurable range of monitoring the target structure, compared to the existing methods. The following Table 3 shows the comparison in terms of available measurement range, sampling rate allowed, and effective resolution.

The existing approaches monitor the displacement for the static target on the static spatial plane, but our approach introduces a case study for the multiple markers on the structures, which are distributed at various distance of 5~72 m. The individual planes on multiple markers are sequentially projected to the standard plane of the PTZ-based vision system based on the proposed distortion cancellation technique. Our approach considers monitoring all markers with one PTZ camera, so that the total sample rate in monitoring the target will be decreased, but this drawback can be ignored due to extremely slow movement of the building structure as an assumption.

There are also various factors causing additional errors in the measurement result compared to the actual displacement, which is manually controlled by the test environment and measured by the conventional distance-measuring instrument. Considering application-specific requirements, we assume that the proposed system recognizes the centimeter-order movement on the target wall. Our experimental environment could measure displacement at the millimeter scale. The camera calibration, which reduces the errors caused by the lens distortion, has to be performed for more accurate measurement at specific zoom levels, distances, and angles for the multiple targets. The experiment is done for multiple targets at various distance and with a wide range of angles. The current status of this approach is still lacking with regards to providing a statistical analysis and calibration by the camera lens distortion. This paper focuses on introducing the feasibility of displacement measurement using a PTZ-based vision system. More analysis of the quantitative relationship causing the measurement errors is needed to improve the total accuracy and resolution in future work.

## 4. Conclusions

In this paper, we propose a PTZ camera-based displacement measurement system for the early detection of building destruction. Our study focuses on extending the measurable range and measuring displacement accurately. In this system, markers, which are attached on the main parts of the building, are used to represent the low frequency movement of the building. By using the PTZ camera, we can automatically acquire the marker images sequentially in predetermined order in the preset data and extend the measurable range. The acquired image is transmitted to the host computer wirelessly after the algorithm presented in this paper is applied. The first step of the applied algorithm is to find the ROI of the marker in the image using the template matching method. In the ROI, the RANSAC Ellipse fitting algorithm was applied to extract five feature points at sub-pixel resolution. The homography was calculated using extracted feature points and perspective distortion was removed by mapping to a standard plane using the homography. By accumulating the offsets from a previously measured marker point to the currently measured marker point in the standard plane, the displacement can be calculated. In order to verify the performance of the proposed system, nine markers are attached to three buildings for which the proposed algorithm is applied. Whereas existing papers measure only one plane in the range 2.5~25 m, our approach could measure more than nine planes in the range 5~72 m, still providing measurement accuracy (RMS) of 0.05~0.26 mm.

## Figures and Tables

**Table 1:** Marker information.

Marker	Structure (Building No.)	Distance from Camera (Installed; m)
1	A	5
2	A	8
3	B	15
4	B	20
5	B	25
6	C	43
7	C	51
8	C	62
9	C	72

**Table 2:** Spatial resolution and displacement measurement according to the distance.

Marker	Distance (m)	FOV (W mm × H mm)	Zoom	mm/Pixel	Root Mean Square (*RMS*)	Max Error	Min Error
1	5	854 × 480	2×	0.445	0.046	0.068	<0.001
2	8	847 × 476	5×	0.441	0.078	0.117	<0.001
3	15	847 × 477	10×	0.441	0.099	0.148	<0.001
4	20	849 × 478	17×	0.442	0.073	0.110	<0.001
5	25	841 × 473	21×	0.438	0.163	0.245	<0.001
6	43	915 × 515	30×	0.477	0.099	0.149	0.002
7	51	1162 × 653	32×	0.605	0.226	0.339	0.004
8	62	1355 × 762	32×	0.706	0.234	0.351	0.001
9	72	1698 × 955	32×	0.885	0.262	0.393	0.002

**Table 3:** Comparison to existing methods.

Method	Measurable Range	Sample Rate (Frame/s)	*RMS* (mm)
[7]	one plane, 8 m away	150	0.18–0.39
[8]	one plane, 2.5 m away	N/A	0.063
[9]	one plane, 5–25 m away	64–500	0.067–0.274
[10]	one plane, 5–20 m away	30	0.060–0.671
[19]	one plane, 2–9 m away	60	0.057–0.661
our method	over nine planes, 5–72 m away	60 (1 s for one plane)	0.05–0.26

## References

[1] Park J.W., Lee J.J., Jung H.J., Myung H. (2010). Vision-based displacement measurement method for high-rise building structures using partitioning approach. NDT E Int..

[2] Kim S.W., Kim N.S. (2013). Dynamic characteristics of suspension bridge hanger cables using digital image processing. NDT E Int..

[3] Lee J.J., Shinozuka M. (2006). A vision-based system for remote sensing of bridge displacement. NDT E Int..

[4] Pan B., Tian L., Song X. (2016). Real-time, non-contact and targetless measurement of vertical deflection of bridges using off-axis digital image correlation. NDT E Int..

[5] Stephen G., Brownjohn J., Taylor C. (1993). Measurements of static and dynamic displacement from visual monitoring of the Humber Bridge. Eng. Struct..

[6] Kohut P., Holak K., Martowicz A. (2012). An uncertainty propagation in developed vision based measurement system aided by numerical and experimental tests. J. Theor. Appl. Mech..

[7] Feng D., Feng M.Q., Ozer E., Fukuda Y. (2015). A Vision-Based Sensor for Noncontact Structural Displacement Measurement. Sensors.

[8] Sładek J., Ostrowska K., Kohut P., Holak K., Gąska A., Uhl T. (2013). Development of a vision based deflection measurement system and its accuracy assessment. Measurement.

[9] Ribeiro D., Calçada R., Ferreira J., Martins T. (2014). Non-contact measurement of the dynamic displacement of railway bridges using an advanced video-based system. Eng. Struct..

[10] Fukuda Y., Feng M.Q., Shinozuka M. (2010). Cost-effective vision-based system for monitoring dynamic response of civil engineering structures. Struct. Control Health Monit..

[11] Lee J.J., Ho H.N., Lee J.H. (2012). A Vision-Based Dynamic Rotational Angle Measurement System for Large Civil Structures. Sensors.

[12] Feng D., Feng M.Q. (2017). Experimental validation of cost-effective vision-based structural health monitoring. Mech. Syst. Signal Process..

[13] Feng D., Scarangello T., Feng M.Q., Ye Q. (2017). Cable tension force estimate using novel noncontact vision-based sensor. Measurement.

[14] Otsu N. (1979). A Threshold Selection Method from Gray-Level Histograms. IEEE Trans. Syst. Man Cybern..

[15] Bolles R.C., Fischler M.A. (1981). A RANSAC-based Approach to Model Fitting and Its Application to Finding Cylinders in Range Data. Proceedings of the 7th International Joint Conference on Artificial Intelligence, IJCAI’81.

[16] Hartley R.I., Zisserman A. (2004). Multiple View Geometry in Computer Vision.

[17] Abdel-Aziz Y., Karara H., Hauck M. (2015). Direct Linear Transformation from Comparator Coordinates into Object Space Coordinates in Close-Range Photogrammetry. Photogramm. Eng. Remote Sens..

[18] Kuruppu G., Manoj C., Kodituwakku S.R., Pinidiyaarachchi U.A.J. Comparison of different template matching algorithms in high speed sports motion tracking. Proceedings of the 2013 IEEE 8th International Conference on Industrial and Information Systems.

[19] Choi H.S., Cheung J.H., Kim S.H., Ahn J.H. (2011). Structural dynamic displacement vision system using digital image processing. NDT E Int..

